# Death in the Tooth-brush

**Published:** 1894-07

**Authors:** 


DEATH IN THE TOOTH-BRUSH.—*Il faut souffrir pour être belle,* say the French, and such as have any teeth of their own left must not spare the tooth-brush, though it excoriate the gums and leave its bristles sticking in unsuspected corners of the mouth "like quills upon a fretful porcupine." Most of us are content to grin and bear these minor miseries of the toilet with such grace as we may, while seeking perseveringly for the ideal tooth-brush, which is as elusive as the philosopher's stone. A serious view of the matter, however, is suggested by a case recently reported in an American journal, in which an operation for appendicitis is said to have revealed the fact that the disease was due to the presence of tooth-brush bristles in the vermiform appendix. The operator, who practices in Albany, expressed the opinion that these "unconsidered trifles" are responsible for many obscure throat, stomach, and intestinal ailments. The moral appears to be that it is an ill-judged economy to use cheap tooth-brushes in which the bristles are simply glued on, and that, after the ordinary ceremony of tooth-cleaning has been gone through, a subsequent "lustration" of the mouth is advisable for the removal of migratory bristles.—*British Medical Journal. The Amer. Pract. and News.*

